# Synthesis and bioactivities of silver nanoparticles capped with 5-Amino-?-resorcylic acid hydrochloride dihydrate

**DOI:** 10.1186/s12951-014-0034-8

**Published:** 2014-09-09

**Authors:** Syeda Sohaila Naz, Muhammad Raza Shah, Nazar Ul Islam, Ajmal Khan, Samina Nazir, Sara Qaisar, Syed Sartaj Alam

**Affiliations:** 1 Nanoscience and Catalysis Division, National Centre for Physics, Quaid-i-Azam University Campus, Islamabad 44000, Pakistan; 2 H.E.J. Research Institute of Chemistry, International Centre for Chemical and Biological Sciences, University of Karachi, Karachi 75270, Pakistan; 3 Institute of Chemical Sciences, University of Peshawar, Peshawar 25120, Pakistan; 4 Sarhad University of Science and Information Technology, Peshawar 2500, Pakistan; 5 Department of Plant Pathology, Khyber Pakhtunkhwa Agricultural University Peshawar, Peshawar, Pakistan

**Keywords:** 5-Amino-?-resorcylic acid hydrochloride dihydrate, Silver nanoparticles, Urease, Xanthine oxidase

## Abstract

**Background:**

Conjugated and drug loaded silver nanoparticles are getting an increased attention for various biomedical applications. Nanoconjugates showed significant enhancement in biological activity in comparison to free drug molecules. In this perspective, we report the synthesis of bioactive silver capped with 5-Amino-?-resorcylic acid hydrochloride dihydrate (AR). The *in vitro* antimicrobial (antibacterial, antifungal), enzyme inhibition (xanthine oxidase, urease, carbonic anhydrase, ?-chymotrypsin, cholinesterase) and antioxidant activities of the developed nanostructures was investigated before and after conjugation to silver metal.

**Results:**

The conjugation of AR to silver was confirmed through FTIR, UV¿vis and TEM techniques. The amount of AR conjugated with silver was characterized through UV¿vis spectroscopy and found to be 9% by weight. The stability of synthesized nanoconjugates against temperature, high salt concentration and pH was found to be good. Nanoconjugates, showed significant synergic enzyme inhibition effect against xanthine and urease enzymes in comparison to standard drugs, pure ligand and silver.

**Conclusions:**

Our synthesized nanoconjugate was found be to efficient selective xanthine and urease inhibitors in comparison to Ag and AR. On a per weight basis, our nanoconjugates required less amount of AR (about 11 times) for inhibition of these enzymes.

## Background

In the new research areas of science, the research field of metal nanoparticles is an extensive and the most emerging field. The huge surface area of the metal nanoparticles is responsible for their diverse optical, chemical, magnetic, mechanical, and catalytic properties as compared to large bulk materials [1],[2]. Different methods like the chemical, physical, and biological methods are reported for the metal nanoparticles synthesis [3]¿[8]. Silver nanoparticles (AgNPs) have been synthesized using various methods, i.e. polysaccharide method, tollens method, irradiation method, biological, polyoxometalates. Silver is well-known for its antimicrobial activities and has been utilized for years towards biomedical applications [9].

Current research showed that conjugation of Ag to plant extracts boosted the enzyme inhibition and antimicrobial activities but these reports are not very systematic [10],[11]. In this context the aim of our present work is to synthesize AgNPs from a synthetic biocide 5-Amino-?-resorcylic acid hydrochloride dihydrate (AR). The choice of AR towards facile synthesis of AgNPs seems to be original one and may present several advantages in terms of water solubility, possibility to conjugation to Ag metal due to the presence of carboxylic, amino and phenolic hydroxyl groups. To ascertain the potential of the synthesized nanoconjugates for *in vivo* applications, the stability of the suspensions was investigated against several parameters such as pH, temperature and salt concentration. Barron AgNPs (Ag) was prepared by reduction of AgNO_3_ with NaBH_4_. The *in vitro* antibacterial, antifungal, enzyme inhibition (xanthine oxidase, urease, carbonic anhydrase, ?-chymotrypsin, cholinesterase) and antioxidant activities of AgAR nanoconjugates were compared with pure AR, Ag and the commercially available antibiotics, enzyme inhibitors and antioxidants.

## Results and discussion

**Figure 1:**
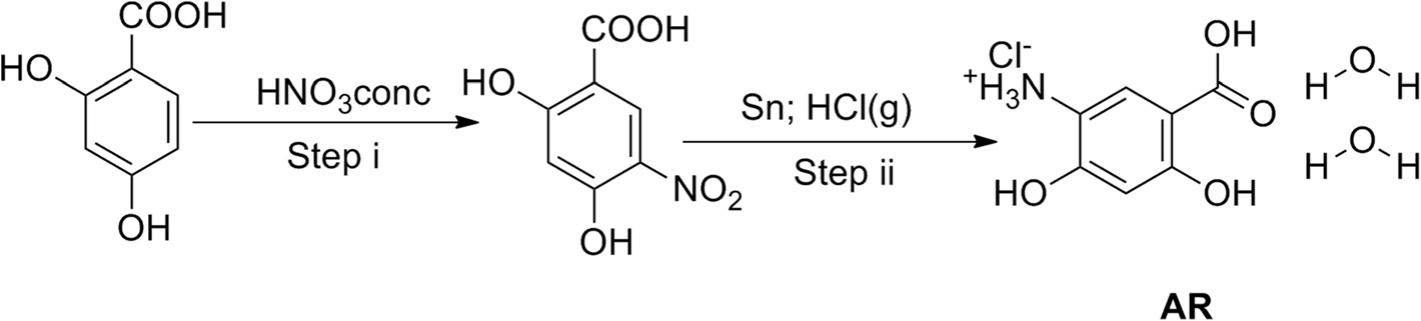
Synthesis of 5-Amino-?-resorcylic acid hydrochloride dihydrate (AR).

**Figure 2:**
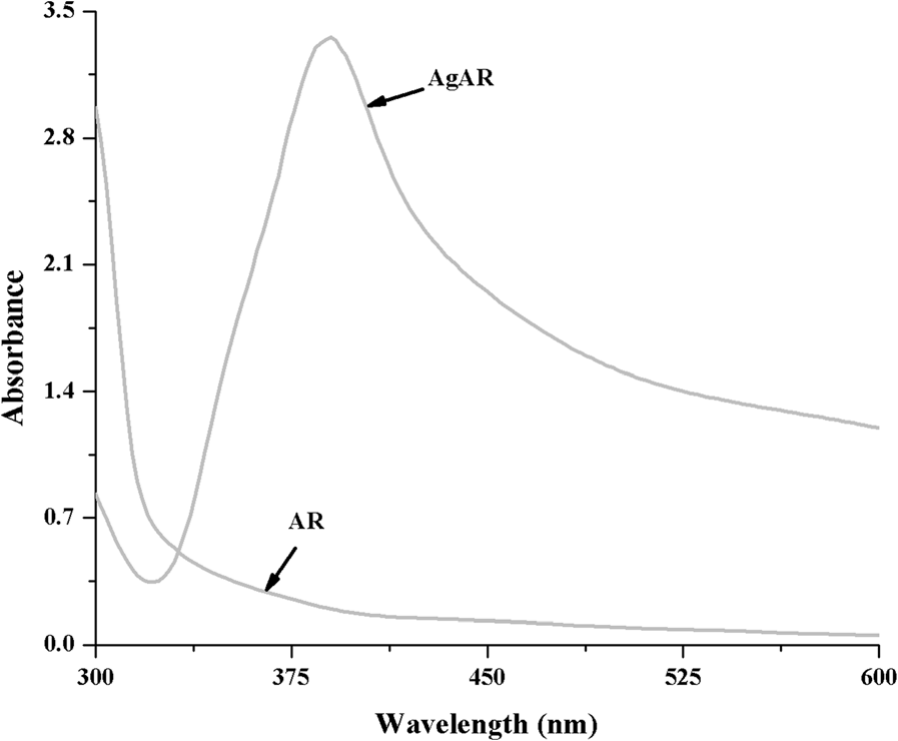
Comparative UV¿vis spectra of AR and AgAR.

The synthesis of AR (Figure 1) was carried out according to our previously published procedure [12]. When the synthesized AR was added to the aqueous solution AgNO_3_, we observed a change in color from light brown to dark brown upon slow addition of NaBH_4_ (Additional file 1: Figure S1). Characterization of AgNPs with UV¿vis spectroscopy showed surface plasmon resonance peak at 390 nm and the amount of AR conjugated with the surface of silver was found to be 9% by weight (Figure 2).

**Figure 3:**
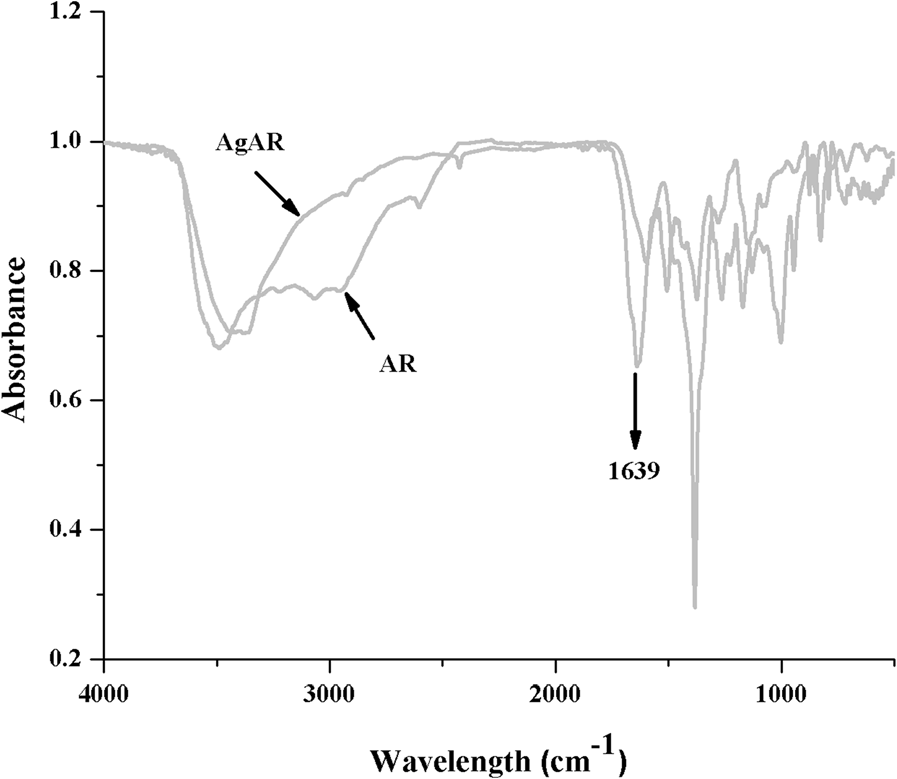
Comparative FTIR spectra of AR and AgAR.

**Figure 4:**
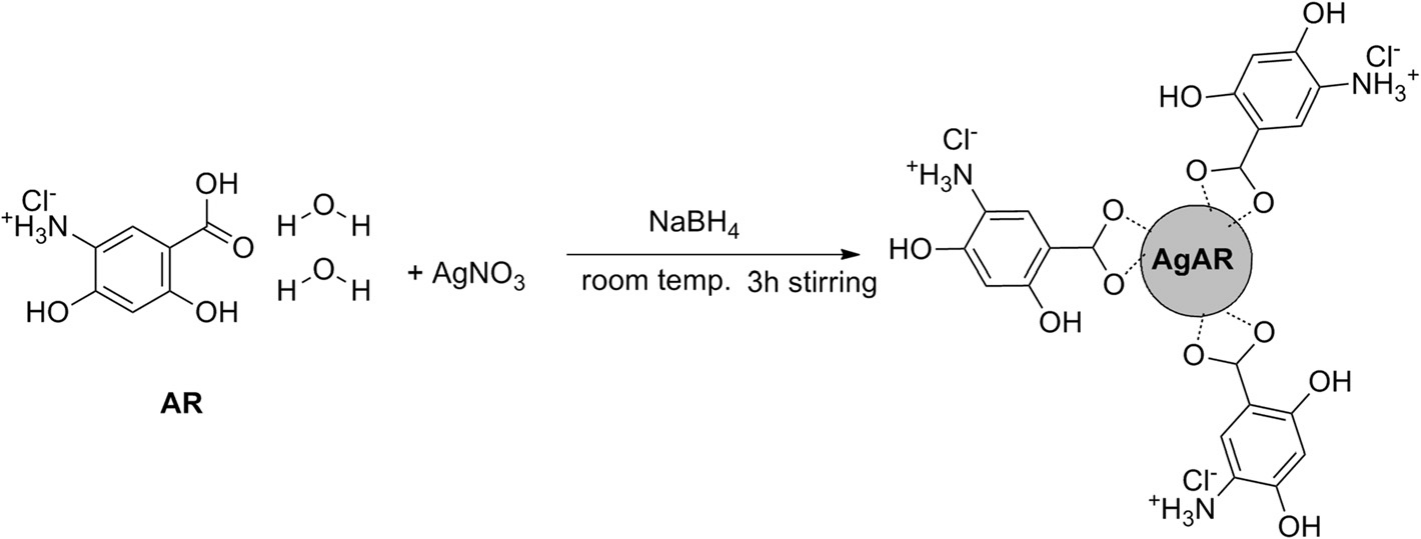
Mechanism of synthesis of silver nanoparticles (AgAR) from AR.

**Figure 5:**
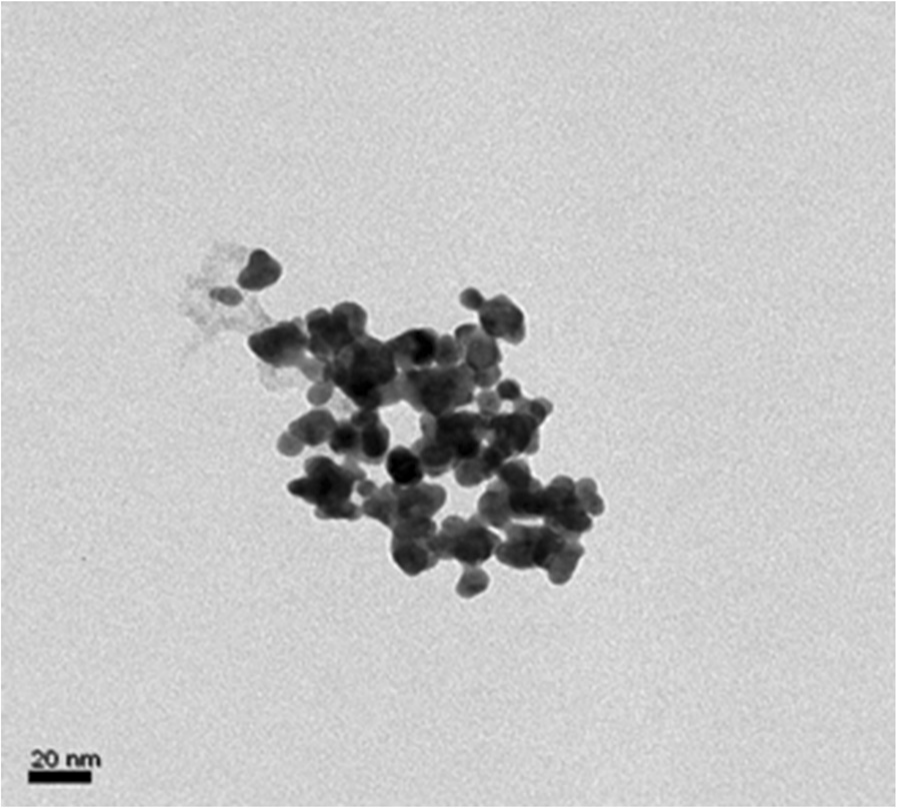
Typical TEM image of AgAR.

FTIR spectra of AR was recorded before and after formation of nanoparticles and reported in Figure 3. The disappearance of the peak at carbonyl region (1639 cm^?1^) in the spectrum of AR indicated the chelation of carboxylic group with silver. From FTIR characterization, a mechanism has been proposed for the synthesis of AgAR nanoconjugates and reported in Figure 4. This figure showed that NaBH_4_ has been involved in reduction of AgNO_3_ while carboxylic group of AR provide stability to AgNPs *via* electrostatic interactions [13]. The formation of silver nanoparticles was finally confirmed from transmission electron micrograph and the mean size of the nanoparticles was found to be 8 nm (Figure 5).

**Figure 6:**
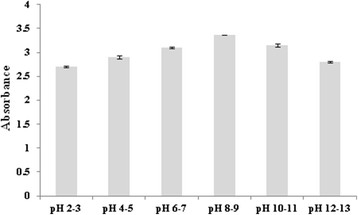
**Effect of pH on stability of AgAR: After 24 h.** Error bars indicate S.D (n = 3).

In order to determine the potential of synthesized nanoparticles for *in vivo* applications, it was desired to check its stability against high concentration of NaCl, heat and pH. The synthesized nanoconjugates was found to be basic in nature as its pH was found to be 8.49. The stability of nanoparticles was checked at all pH values ranging from 2¿13 (Figure 6) and indicated by observing a change in ?_max_. In comparison to other pH values, as the absorbance of nanoparticles was highest at pH 8¿9 therefore, it was established that the stability of the nanoconjugates was good at this pH.

**Figure 7:**
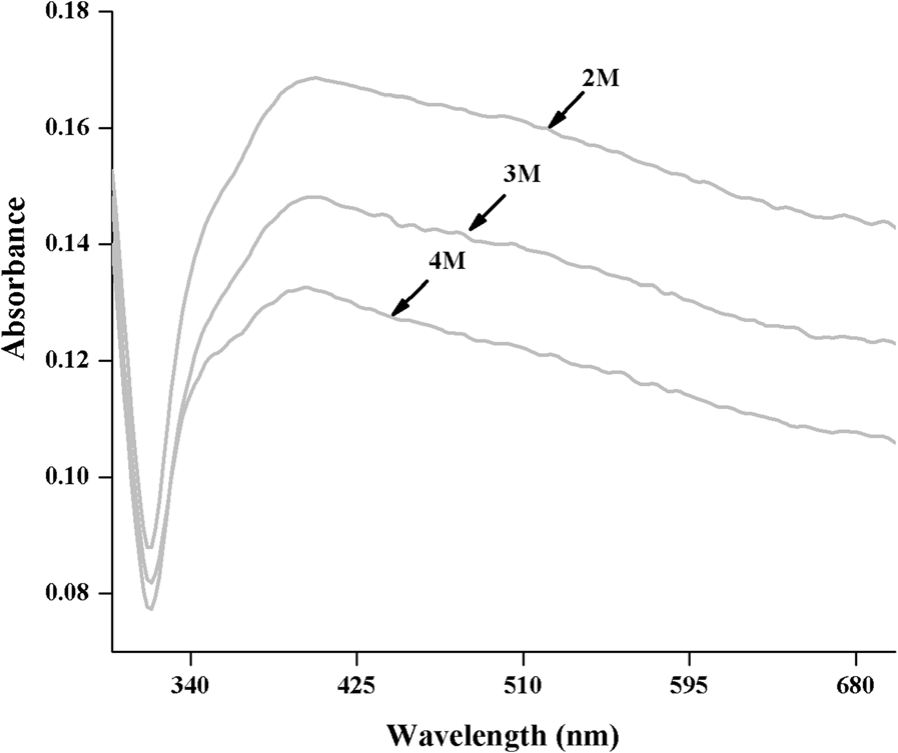
Effect of salt (NaCl) on stability AgAR: After 24 h.

When NaCl was added to the nanoparticles solution, a gradual change in the peak shape is observed; an initial halide surface layer of unknown structure may form very rapidly (Figure 7). The successive changes in the UV-visible spectra proposed that this layer may have developed into a silver halide layer. For NaCl, the onset concentration for aggregation is considerably lower. This has been discussed in terms of a distinct effect on the nanoparticles surface, in which the surface charge is dropped by nearly a factor of 2. It is not clear that how this is accomplished. One probability is that a chloride layer decreased the number of adsorption sites for the highly charged AR. Instead, the chloride ion may substitute AR entirely but then form AgCl_2_ rather than AgCl, thereby retaining a negatively charged surface but with a lesser value [14].

**Figure 8:**
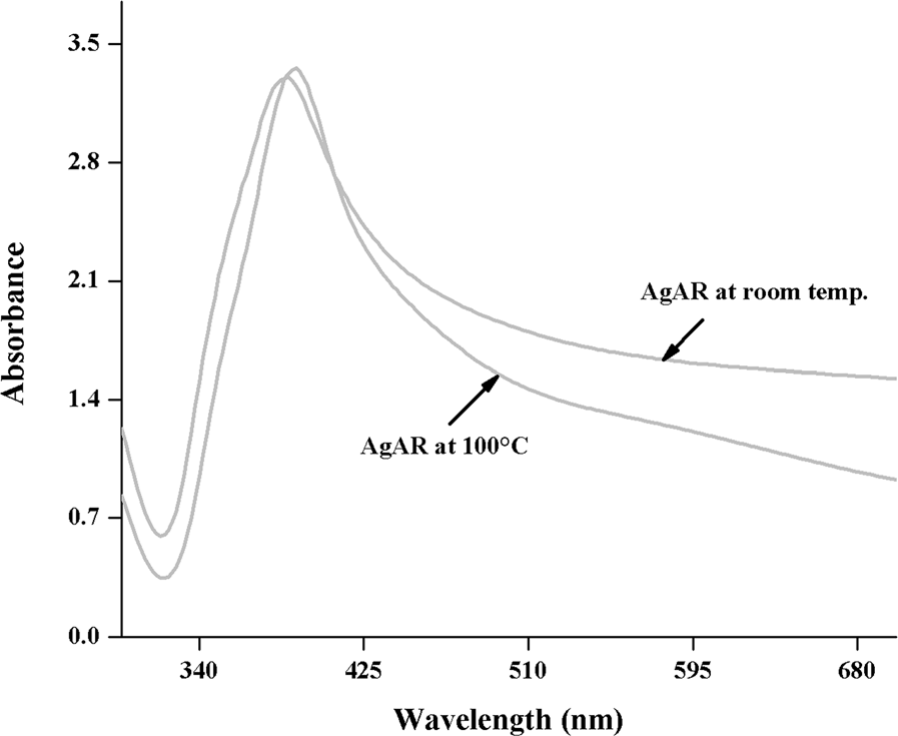
Effect of heat on stability of AgNPs stabilize with AR: After 24 h.

Figure 8 showed the absorption spectra of 8 nm AgNPs at 100°C. The result indicated that the temperature effect is negligible, resulting in a very minute reduction in absorbance while a broadening of the plasmon band was not observed.

In the present study, a systematic comparative bioactivity evaluation of synthesized AR, Ag, AgAR and standard drugs was carried out. AgAR nanoconjugates contain 9% of AR as determine from UV¿vis study. The purpose of the conjugation of AR to the surface of AgNPs is to determine the change in the activity of AR after attachment to the surface of metal. Selected bioassays include *in vitro* antimicrobial activities, six enzyme inhibition activities and antioxidant activities.

**Figure 9:**
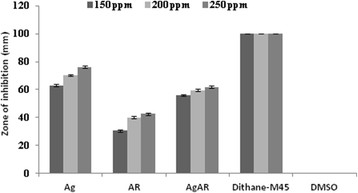
**Antibacterial activity of Ag, AR, AgAR, streptomycin (standard drug) and DMSO (negative control) against*E. carotovora*.** Error bars indicate S.D (n = 3).

**Figure 10:**
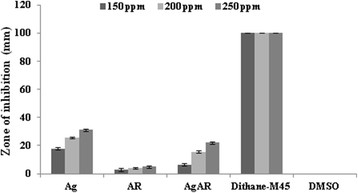
**Antifungal activity of Ag, AR, AgAR, dithane-M45 (standard drug) and DMSO (negative control) against*A. solani.*** Each value is the mean ± S.D (n = 3).

**Figure 11:**
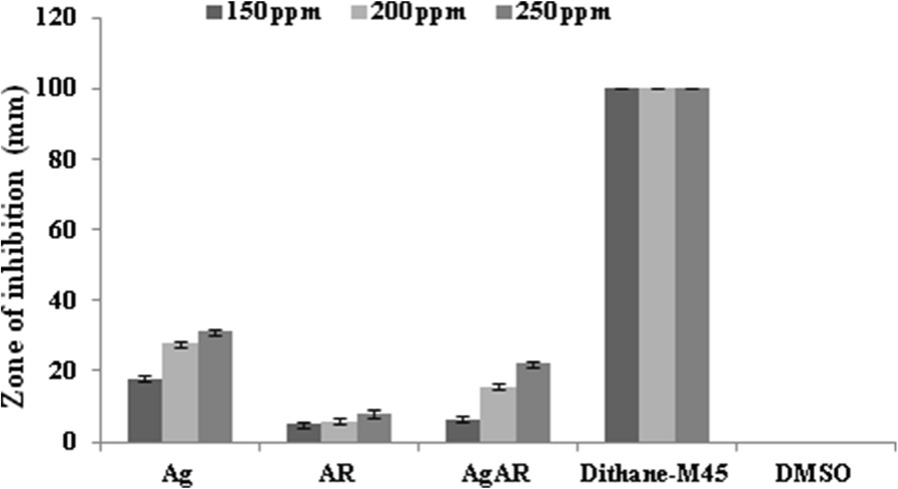
**Antifungal activity of Ag, AR, AgAR, dithane-M45 (standard drug) and DMSO (negative control) against*F. solani.*** Error bars indicate S.D (n = 3).

The antimicrobial studies of Ag, AR and AgAR were determined against three pathogenic microbes namely *Erwinia carotovora* (Figure 9)*, Alternaria solani* (Figure 10) *and Fusarium solani* (Figure 11) at three different concentrations (150, 200 and 250 ppm)*.* Silver in the form of bare AgNPs (Ag) showed significant antibacterial activity in comparison to AR and AgAR. However, at the tested concentrations the activity of Ag was lesser than the standard antibiotic streptomycin. Moreover, the antifungal activities of Ag, AR and AgAR were not significant in comparison to the standard fungicide dithane-M45.

The synthesized nanoparticles AgAR, Ag and parent compound AR were then screened for inhibition of xanthine and urease enzymes and the results are reported in Table 1. Ag and AR are found to be inactive against these enzymes. The AgAR has shown significant inhibition against xanthine oxidase and urease enzymes with IC_50_ value of 15.5 ± 0.9 and 9.2 ± 0.9 ?g/mL, respectively. As discussed previously our nanoconjugates contains 9% of ligand AR, therefore the conjugation of AR to Ag in the form of nanoconjugates AgAR not only enhanced the enzyme inhibition activity of AR but also reduced the amount of AR by about 11 times. Moreover, the urease inhibition activity of AgAR was found to be more than the standard drug thiourea with IC_50_ values of 9.2 ± 0.9 and 21.8 ± 1.2 ?g/mL, respectively. Its means the conjugation of AR to Ag had a robust inhibition effect in comparison to pure Ag and AR. In order to evaluate the selectivity of AgAR towards xanthine oxidase and urease enzymes, the activity of AgAR, Ag and AR were also tested against the carbonic anhydrase, ?-chymotrypsin, acetylcholinesterase and butylcholinesterase enzymes. Interestingly the AgAR, Ag and AR were found to be inactive against these enzymes, exhibiting their selectivity towards xanthine oxidase and urease enzymes. In case of carbonic anhydrase AR showed inhibitory effect against carbonic anhydrase-II, but this effect was non-significant in comparison to standard drug acetazolamide.

**Table 1:** Comparative enzyme inhibition activities of AR, Ag and AgAR

**S. No**	**Enzymes**		**AR**	**Ag**	**AgAR**	**Standard**
1	Xanthine oxidase	% Inhibition	8	9.5	86.8	98.6
		IC_50_ ± S.E.M* (?g/mL)	°NA	NA	15.5 ± 0.9	2.0 ± 0.1
2	Carbonic anhydrase-II	% Inhibition	71.2	20	0	89.0
		IC_50_ ± S.E.M (?g/mL)	23.43 ± 1.12	NA	NA	0.21 ± 0.03
3	*?*-Chymotrypsin	% Inhibition	7	10	0	98.6
		IC_50_ ± S.E.M (?g/mL)	NA	NA	NA	5.7 ± 0.1
4	Acetylcholi-nesterase	% Inhibition	8	5	0	98.6
		IC_50_ ± S.E.M (?g/mL)	NA	NA	NA	8.5 ± 0.1
5	Butylcholin-esterase	% Inhibition	12	8	0	98.6
		IC_50_ ± S.E.M (?g/mL)	NA	NA	NA	8.5 ± 0.1
6	Urease	% Inhibition	5	15	97.0	96.9
		IC_50_ ± S.E.M(?g/mL	NA	NA	9.2 ± 0.9	21.8 ± 1.2

°NA = Not applicable, *S.E.M = Standard error mean.

The antioxidant activity of AgAR, Ag and AR was determined using DPPH radical scavenging assay (RSA). Before conjugation, the antioxidant activity of AR was significant with IC_50_ value of 36.55 ± 2.05 ?g/mL but after conjugation the AR lost its antioxidant activity (Table 2). Additionally, the antioxidant effect produced by AR in comparison to AgAR was also greater than all the three standard drugs (ascorbic acid, butylated hydroxyanisole, N-acetyl cystine).

**Table 2:** DPPH radical scavenging activities of Ag, AR and AgAR

**S. No.**	**Code**	**IC**_**50**_**± S.E.M (?g/mL)**	RSA*(%)
1	AR	36.55 ± 2.05	93
2	Ag	NA	10
3	AgAR	NA	5
4	AA¿	40.1 ± 1.2	96
5	BHA¿	44.6 ± 0.7	95
6	NAC^»^	105.9 ± 1.1	95

*Radical scavenger activity, ¿AA (ascorbic acid), ¿BHA (butylated hydroxyanisole), ^»^NAC (N-acetyl cystine).

## Conclusions

In conclusion, we have developed an efficient strategy for the synthesis of silver nanoparticles capped with AR toward selective inhibition of urease and xanthine enzymes. The urease and xanthine inhibition potency of AR was amplified by conjugation to Ag. On per weight basis, our nanoconjugates AgAR require less amount of AR (about 11 times) for inhibition enzyme of urease and xanthine enzymes. Moreover, our synthesized nanoconjugates was found to be stable at all pH vales and was not affected by high concentration of salt solution and heating up to 100°C.

## Methods

### Materials and instruments

Silver nitrate (AgNO_3_), sodium borohydride (NaBH_4_), sodium hydroxide (NaOH), sodium chloride (NaCl), methanol (MeOH), dimethylformamide (DMF) and hydrochloric acid (HCl) were purchased from Merck. Deionized water was used for synthesis of AgNPs. A digital pH meter model 510 (Oakton, Eutech) equipped with a glass working electrode and a reference Ag/AgCl electrode was used. UV¿vis spectra were recorded with a Shimadzu UV-240, Hitachi U-3200 spectrometer with a path length of 1 cm. The FTIR spectra were recorded using Shimadzu IR. Microscopic image of synthesized AgNPs was taken with a Zeiss Libra transmission electron microscope (TEM) operated at 120 keV.

### Synthesis of ligand and nanoparticles

#### Synthesis of 5-Amino-?-resorcylic acid hydrochloride dihydrate (AR)

Synthesis of AR illustrated in Figure 1 was carried out by our previously reported method [12]. It was used further as a capping agent for silver nanoparticles.

#### Synthesis of Silver nanoparticles capped with AR (AgAR)

As AR has low solubility in water therefore a mixture of MeOH, DMF and water (2:3:5) was used as a solvent for its solubility. 1 mM solution of AR was prepared in respective solvent and used for synthesis of AgNPs. 1 mM solution of AgNO_3_ was prepared in deionized water. Freshly prepared (40 mM in water) solution of NaBH_4_ was used as a reducing agent. I mL of AR and 10 mL of Ag NO_3_ (1 mM) was taken and kept on stirring for 30 min. Then 1 mL of NaBH_4_ was added drop wise. After addition of reducing agent, the color of the reaction mixture generally changed from light brown to dark brown (Additional file 1: Figure S1). After 3 h stirring, the solution was characterized by UV¿vis spectroscopy (Figure 1). Similarly different ratios of Ag, AR and NaBH_4_ were tested (Additional file 2: Table S1). From table S1 the optimum ratio for nanoparticles formation was found to be 10: 1.0: 1.0 (Ag: AR: NaBH_4_). Solid silver nanoparticles capped with AR (AgAR) were collected by freeze drying for FTIR and TEM characterizations and bioactivity screening.

#### Bare silver nanoparticles (Ag)

10 mL of Ag NO_3_ (1 mM) was taken followed by drop wise addition of NaBH_4_ and then kept on stirring for 30 min. Solid bare silver nanoparticles (Ag) was collected after freeze drying.

### Stability check of silver nanoparticles

#### pH effect

For pH study, 2 mL of freshly prepared AgAR was taken. Its pH was recorded and found to be 8.49. The pH of AgAR ranging from 10¿13 was adjusted by using 1 M NaOH solution. Similarly, the pH of AgAR ranging from 2¿7 was maintained by using 1 N HCl. The UV¿vis spectra of resulting solutions were recorded after 24 h (Figure 6).

#### Salt effect

Effect of high concentration of NaCl (2¿4 M) on synthesized AgAR was studied. For this purpose 2 mL of freshly prepared AgAR was taken in a beaker. Then 2 mL of 2 M NaCl solution was added to it. The resulting solution was kept at room temperature for 24 h. Then its UV¿vis spectrum was recorded. Similarly, the effect of 3 and 4 M NaCl was monitored by following the same protocol (Figure 7).

#### Heat effect

Heat effect of synthesized nanoparticles AgAR was studied by taking 10 mL of freshly prepared AgAR in a round bottom flask (25 mL). The solution was heated up to 100°C for 30 min. The nanoparticles was then kept at room temperature for and its UV¿vis spectrum was recorded (Figure 8).

### Bioactivity screening of silver nanoparticles

#### Antifungal activity

The antifungal activity was evaluated by the agar-well diffusion method [15]. Dithane-M45 was used as a standard drug. Three different concentration (150, 200 and 250 ppm) of standard drug and tested samples prepared in dimethysulfoxide (DMSO). 10 ?L of each sample was uniformly dissolved in PDA (potato dextrose agar) medium on agar plate. Using sterilized borer mycelial plug (5 mm) of *Alternaria solani* and *Fusarium oxysporum* f. sp. *lycopersici* (FOL) were fixed at the center of the agar plate. The positive control (Dithane-M45 fungicide) and negative control (media?+?DMSO) were also run in parallel for reference. The agar plate cultures were incubated at 25°C for 7 days and growth of the fungal strains were observed on daily basis. After incubation, the cultures were compared for reduction in radial colony growth of the fungus in negative, positive and test cultures. Percent growth Inhibition in radial colony growth was determined in triplicate by using the following formula.

(1) $$I\;=\;\left(C\;?\;T\right)/C\;\times \;100$$

I = Percentage of inhibition, C = Diameter of fungal colony in control, T = Diameter of the fungal colony in treatment.

#### Antibacterial activity

The antibacterial activity of samples was determined according to method described by Yin *et al.*[16]. For this study gram-negative bacteria *Erwinia carotovora* was selected [17]. Streptomycin was used as a positive control. Three different concentrations (150, 200 and 250 ppm) of each sample and standard drug was prepared in DMSO. In sterilized petridishes, 10 mL PDA was poured and bacterial inoculation was carried out by streaking to form different colonies. The disc of 5 mm was kept at the center of agar plate and 10 ?L of each sample was poured on disk. The positive control and negative control (media?+?DMSO) were also run in parallel for reference. and zone of inhibition was observed after 72 h. Percent growth of inhibition was calculated by following formula and analysis was done in triplicate.

(2) $$\mathrm{IE}\;\left(\%\right)\;=\;\mathrm{DC}?\mathrm{DS}/\mathrm{DC}\;¿\;5\;\times \;100$$

DC stands for diameter of control and 5 is the size of disc which was 5 mm.

#### Xanthine oxidase inhibition assay

The XO inhibitory activity of test compounds was determined by measuring the rate of hydroxylation of the substrate (xanthine) and subsequent formation of uric acid, which is a colorless end product of the reaction and showed absorption at 295 nm. Briefly, the reaction mixture containing 10 ?L of 1 mg/L pure compound or 0.2 mg/mL of nanoparticles was dissolved in DMSO, 150 ?L of phosphate buffer (0.05 mol/L, pH 7.4), 0.003 units of Xanthine Oxidase dissolved in buffer (20 ?L), and 20 ?L of 0.1 mmol/L xanthine as substrate for enzyme. After addition of xanthine oxidase, the mixture was incubated for 10 min at room temperature and pre-read in the UV region (*?*_max_ 295 nm). The substrate was added to reaction mixture, and continuous reading for 15 min at an interval of 1 min was observed (Spectra MAX-340). The percentage inhibitory activity induced by the samples were determined against a DMSO blank and calculated using the following formula. Inhibition (%) = 100 - [(OD test compound/OD control)?×?100]. The IC_50_ of the compounds as calculated using EZ-Fit windows-based software (Perrella Scientific Inc. Amherst, U.S.A.). To compare the inhibitory activities of the compounds, allopurinol was used as standard. The reaction for each compound was performed in triplicate [18].

#### Urease inhibition assay

Exact 25 ?L of enzyme (jack bean urease) solution and 5 ?L of test compounds (0.2 mg/mL of nanoparticles) were incubated with 55 ?L of buffers containing 100 mM urea for 15 min at 30°C in each well of 96-well plates,. Ammonia production was measured as a urease activity by indophenol method [19]. Final volumes were maintained as 200 ?L by adding 45 ?L phenol reagent (1% w/v phenol and 0.005% w/v sodium nitroprussside) and 70 ?L of alkali reagent (0.5% w/v NaOH and 0.1% active chloride NaOCl) to each well. Using a microplate reader (Molecular Devices, CA, USA), the increase in absorbance was measured at 630 nm after 50 min at pH 6.8. The results (change in absorbance per min) were collected using softMax Pro software (Molecular Devices, CA, USA). Thiourea was used as the standard inhibitor and percentage inhibitions were calculated as follow: 100-(OD_testwell_/OD_control_)?×?100 [20]. The analysis was done in triplicate.

#### Carbonic anhydrase-II inhibition assay

The experiment was run with the buffer containing HEPES-*Tris* solution at a total concentration of 20 mM and pH (7.2-7.9). For this 140 ?L of the HEPES-*Tris* solution was mixed with 20 ?L of freshly prepared aqueous solution of purified bovine erythrocyte CA-II (0.1-0.2 mg/2000 ?L of demonized water for 96-well), Fluka MP Biomedicals. The test compound was dissolved in 10% DMSO, out of this 20 ?L was added in a reaction mixture, followed by the addition of 4-NPA at concentration of 0.8 mM diluted in ethanol [21]. The reaction was initiated by addition of 4-NPA after 15 min incubation of test compound. The compounds were tested in triplicate. In this assay, the reaction was performed by using 96-well plates. To initiate the reaction, the plate was placed in a microplate reader and the amount of reaction product formed was monitored at 1 min interval for 30 min at 400 nm. The reaction temperature was kept between 25¿28°C.

#### ?-Chymotrypsin assay

The inhibitory activity of *?*-chymotrypsin was performed in 50 mM *Tris¿HCl* buffer pH 7.6 with 10 mM CaCl_2_, as mentioned by Cannell *et al*. with the slight modification. The enzyme *?*-chymotrypsin (12 units/mL prepared in buffer mentioned above) with the 0.5 mM test compound prepared in DMSO, was incubated at 30°C for 25 min. The reaction was initiated by the addition of the chromogenic substrate, *N*-succinyl-L-phenylalanine-*p*-nitroaniline (SP*p*NA; 0.4 mM final concentration prepared in the buffer as above). The change in absorbance by release of *p*-nitroanilide was continuously monitored at 410 nm. The positive control without test compound was replaced by DMSO (final concentration 7%). The analysis was performed in triplicate. The percentage of inhibition based upon initial velocity and calculated as [22]:

(3) $$\%\;\mathrm{Inhibition}\;=\;100?\;\left({OD}_{\text{test}}/{OD}_{\text{control}}\right)*100$$

#### Cholinesterase Inhibition assays

Acetyl cholinesterase (AChE) and butyl cholinesterase (BChE) inhibiting activities were measured by the spectrophotometric method by using acetylthiocholine iodide and butyrylthiocholine chloride as substrates. The reaction mixture contained 130 ?L of (100 mM) sodium phosphate buffer (pH 8.0), 20 ?L of DTNB, 10 ?L of tested compound solution and 20 ?L of AChE or BChE solution, which were mixed and incubated for 15 min at 25°C. The reaction was then initiated by the addition of 20 ?L acetylthiocholine or butyrylthiocholine, respectively. The hydrolysis of acetylthiocholine and butyrylthiocholine were monitored at a wavelength of 412 nm (15 min). Absolute ethanol, which becomes 5% in the reaction mixture, was used as solvent for test compounds and the standard inhibitor. All the reactions were performed in triplicate in 96-well microplate using SpectraMax Plus 384 [23],[24].

#### Antioxidant assay

Measurement of superoxide radical scavenging activity was carried out by the modified method used by Ferda [25]. The reaction mixture comprised of 40 mL of 280 mM b-nicotinamide adenine dinucleotide reduced form (NADH), 40 mL of 80 mM nitro blue tetrazolium (NBT), 20 mL phenazine methosulphate (PMS), 10 mL of 0.2 mg/mL sample and 90 mL of 0.1 M phosphate buffer (pH 7.4). Reagents were prepared in buffer solution and the sample in DMSO. The reaction was performed in 96-well microtitre plate at room temperature and absorbance was measured at 560 nm. The formation of superoxide was monitored by measuring the formation of water soluble blue formazan dye. A lower absorbance of reaction mixture indicates a higher scavenging activity of the sample. Percent radical scavenging activity (% RSA) by samples can be determined in comparison with a control.

(4) $$\mathrm{RSA}\;\left(\%\right)\;=\;100¿\left({OD}_{\text{testcompound}}/{OD}_{\mathrm{control}}\right)*100$$

The reaction mixture containing 5 ?L of (0.5 mg/mL) test samples (in DMSO) and 95 mL of DPPH (300 mmol) in ethanol, was taken in a 96-well microtitre plate and incubated in ELISA (multiple reader, Spectra Max ¿ 3400) at 37°C for 30 min. The absorbance was measured at 515 nm. RSA (%) was determined in triplicate by comparison with a DMSO containing controls. Ascorbic acid, butylated hydroxyanisole and N-acetyl cystine were used as the positive controls [26].

## Abbreviations

AR: 5-Amino-?-resorcylic acid hydrochloride dihydrate

AgAR: Silver nanoparticles capped with AR

AgNPs: Silver nanoparticles

UV-Visible spectra: Ultra violet-visible spectra

FTIR: Fourier transformed infrared

TEM: Transmission electron microscope

## Competing interests

The authors state that they have no competing interests.

## Authors¿ contributions

SSN performed the synthesis of nanoparticles and wrote the manuscript. MRS supervised the whole work. AK and SSA helped in interpretation of biological data. NUI, SN and SQ helped with the analysis. All authors read and approved the final manuscript.

## Additional files

## Supplementary Material

Additional file 1: Figure S1. Optical recognition of AgNO_3_, NaBH_4_, AR and AgAR. Click here for file

Additional file 2: Table S1. Optimization of reaction conditions by changing the amount of Ag, AR and NaBH_4_. Click here for file
