# Supplementary figures and images for: Synthesis and bioactivities of silver nanoparticles capped with 5-Amino-?-resorcylic acid hydrochloride dihydrate
Source: J Nanobiotechnology. 2014 Sep 9;12:34. doi: 10.1186/s12951-014-0034-8 (PMC4422292; doi:10.1186/s12951-014-0034-8)

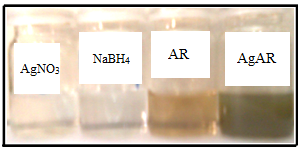

Supplement: Additional file 1: Figure S1. — Optical recognition of AgNO3, NaBH4, AR and AgAR. [file s12951-014-0034-8-S1.tiff]
