# Supplementary material for: Synthesis and bioactivities of silver nanoparticles capped with 5-Amino-?-resorcylic acid hydrochloride dihydrate
Source: J Nanobiotechnology. 2014 Sep 9;12:34. doi: 10.1186/s12951-014-0034-8 (PMC4422292; doi:10.1186/s12951-014-0034-8)
Supplement: Additional file 2: Table S1. — Optimization of reaction conditions by changing the amount of Ag, AR and NaBH4. [file s12951-014-0034-8-S2.pdf]

Table S1 Optimization of reaction conditions by changing the amount of Ag, AR and $NaBH_4$

Sample Code	Ag: AR: $NaBH_4$ (mL)	λ^{max}/abs^{max}(nm)
12,1	12:1:1.2	390/3.095
11,1	11:1:1.1	388/2.586
**10,1**	**10:1:1.0**	**390/3.358**
9,1	9:1:0.9	390/2.654
8,1	8:1:0.8	392/2.726
Conditions: $AgNO_3$ 1 mM; AR 1 mM; $NaBH_4$ 40 mM		
